# Effect of probiotics on digestibility and immunity in infants: A study protocol for a randomized controlled trial

**DOI:** 10.1097/MD.0000000000005953

**Published:** 2017-04-07

**Authors:** Lingli Xiao, Guodong Ding, Yifang Ding, Chaoming Deng, Xiaolei Ze, Liang Chen, Yao Zhang, Lihua Song, Hongli Yan, Fang Liu, Xiaoming Ben

**Affiliations:** a Department of Neonatology, Shanghai First Maternity and Infant Hospital, Tongji University School of Medicine, Shanghai, China; b Department of Pediatrics, Shanghai East Hospital, Tongji University School of Medicine, Shanghai, China; c Biostime Institute for Nutrition and Care, Biostime Co. Ltd., Guangzhou, China; d Department of Food Science and Engineering, Shanghai Jiao Tong University, Shanghai, China; e Department of Laboratory Medicine, Changhai Hospital, Second Military Medical University, Shanghai, China.

**Keywords:** bifidobacteria, infant, probiotics, randomized controlled trial, SIgA

## Abstract

The gastrointestinal (GI) tract of a fetus in utero is sterile but it becomes colonized with environmental microorganisms shortly after birth. Since the gut microbiota undergoes substantial changes in early life, healthy gut microflora is essential to an infant's gut health and immune system and probably also has an effect on overall health status in later life. Probiotics, defined as viable microbial preparations that have a beneficial effect on the health of the host, represent a rapidly expanding field. Although randomized controlled trials using probiotics in infants have shown promising results in the prevention and treatment of common diseases such as diarrhea and allergy, little is known about whether probiotics could offer benefits to healthy infants. We have designed a randomized controlled trial to test the hypothesis that an oral preparation of probiotics is superior to placebo in improving digestive and immune function in healthy infants.

The trial will be a randomized, double-blind, placebo-controlled, 2-parallel-group study in Shanghai, China. After a 2-week run-in period, 200 exclusively formula-fed healthy infants aged 4 to 6 months will be randomly allocated to receive either a probiotic product containing *Bifidobacterium infantis* R0033, *Bifidobacterium bifidum* R0071, and Lactobacillus helveticus R0052 or an identical placebo once daily for 4 weeks and will be followed up for 8 weeks. The duration of the subject's participation will be 14 weeks, with a total of 5 visits: inclusion (Visit 1, Day 1), start of intervention (V2, D15), end of intervention (V3, D44), and follow-up (V4 and V5, D72 and D100). Stool and saliva samples will be collected at the first 3 visits to measure microbial populations and secretory immunoglobulin A (SIgA), respectively. Physical examination will be performed at each visit, and tolerance records will be completed 1 day prior to each visit. The primary endpoints will be the changes in the composition of fecal microbiota, particularly the *Bifidobacterium bifidum* population. The secondary endpoints will include the change in salivary SIgA level, growth parameters, digestive tolerance, and adverse events.

An effective, practical, and acceptable probiotic intervention in manipulating the gut microbiota and boosting the immune system in formula-fed infants would represent a major clinical advance. The administration of probiotic supplementation or follow-on formula to infant may be associated with some clinic benefits.

## 1 Introduction

The gastrointestinal (GI) tract of a normal fetus in utero is generally thought to be sterile. Newborn infants exit the uterus and enter an extrauterine environment filled with microbes. During the birth process and rapidly thereafter, microbes from the mother and the surrounding environment colonize the GI tract of the infant.^[1]^ However, the intestinal microbiota of the infant undergoes substantial development in early life. From an initial low diversity and low complexity, the intestinal microbiota slowly develop and mature, reaching an adult state around 3 years of age.^[2]^ Therefore, the first 2 to 3 years of life are the most critical period in which one can intervene to shape the microbiota as best as possible. Gut-associated immune tissue represents almost 80% of the immune system,^[3]^ making the composition of intestinal microbiota an important factor in the immune system. It is considered that the onset of many diseases possibly relates to disruption of the early colonization and establishment of the gut.^[4]^ All these factors indicate that healthy gut microflora is essential to infant's gut health and immune system and probably also has an effect on overall health status in later life.

Probiotics, defined as viable microbial preparations that have a beneficial effect on the health of the host, represent a rapidly expanding field. The demand for probiotics in clinical applications and as functional foods has been dramatically increasing in spite of limited understanding of their mechanisms. Numerous randomized controlled trials using probiotics in infants have shown promising results in the prevention and treatment of common diseases such as diarrhea and allergy.^[5–8]^ Among the possible mechanisms of probiotic intervention is promotion of a nonimmunologic gut defense barrier, which includes the normalization of increased intestinal permeability and altered gut microecology. Another possible mechanism of probiotic intervention is improvement of the intestine's immunologic barrier, particularly through local immunoglobulin A (IgA) responses and alleviation of intestinal inflammatory responses, which produce a gut-stabilizing effect.^[9]^ However, little is known about whether probiotics could offer benefits to healthy infants.

Among a number of bacterial species of that are found in the GI tract, *Bifidobacterium* and *Lactobacillus* species have been explored extensively in human and animal studies. The 2 species are recognized to have health-promoting properties and are added to commercial foods such as infant formula and used in pharmaceutical probiotics to enhance the protective gut microbiota, thus improving intestinal microbial balance.^[10]^*Bifidobacterium infantis* releases bioactive factors and prevents TNF-α- and IFN-γ-induced drops in transepithelial resistance to protect intestinal epithelial cell barrier function.^[11,12]^*Bifidobacterium bifidum* increases local IgA levels in the intestine and prevents diarrhea and shedding of rotavirus.^[13,14]^*Lactobacillus helveticus* inhibits *Campylobacter jejuni* invasion of intestinal epithelial cells.^[15]^ Its surface-layer protein can exert anti-inflammatory effects by reducing the activation of NF-κB on intestinal epithelial cells.^[16]^

However, the interpretation of clinical trials is difficult to compare due to the differences in endpoints, and variations in the probiotics used, as well as their doses and strains. It is well known that substantial differences exist between different probiotic bacterial species and strains. A recent review has suggested that sufficient evidence is available to warrant further evaluation.^[17]^ Huang et al^[18]^ examined the effect of a probiotic mixture containing *Bifidobacterium infantis*, *Bifidobacterium bifidum*, and *Lactobacillus helveticus* on the intestinal flora in rodent animal models and found that the numbers of bifidobacteria were higher in the probiotic group than those in the control group. Cazzola et al^[19]^ investigated the efficacy of a synbiotic supplementation including the above 3 strains in reducing common winter diseases in children and observed that the supplementation could decrease the risk of occurrence of common infectious diseases.

Therefore, we hypothesize that a combination of the above 3 strains containing *Bifidobacterium infantis*, *Bifidobacterium bifidum*, and *Lactobacillus helveticus* might be beneficially effective on infants’ overall health. Here, we have provided a protocol for a randomized controlled trial to determine the effects of a combination of the novel 3-strain probiotics on digestibility and immunity in healthy infants.

## 2 Materials and methods

### 2.1 Statistical analysis

Statistical analysis of all data will be performed by a specialized statistician in a blind manner. To estimate the efficacy of this trial, both the intention-to-treat and per-protocol populations will be analyzed. Baseline characteristics will be compared by unpaired Student's *t*-test (parametric test) or Mann–Whitney test (nonparametric test) and chi-square or Fisher's exact test (if one of the expected frequency < 5). All statistical analyses of the data will be performed using SAS software version 9.3 or higher (SAS Institute Inc., Cary, NC), and a *P*-value < 0.05 will be considered statistically significant. The target variables for analysis are as follows.

### 2.2 Primary analysis

The primary endpoints, the V2-V3 changes in *Bifidobacterium bifidum*, *Bifidobacterium longum ssp. infantis*, *Lactobacillus helveticus*, *Clostridium perfringens*, *Enterococcus*, *Enterobacteria*, and *Bacteroides* in stools will be analyzed using the following ANCOVA model (SAS PROC MIXED):

*Y* = Product +Baseline + (Product × Baseline)

where

Y = V2–V3 change in fecal microbiota

Product = study products

Baseline = baseline value (V2)

Product × Baseline = Product − Baseline interaction

### 2.3 Secondary analysis

The above-mentioned ANCOVA model will be conducted for analysis of the V2–V3 change in salivary SIgA levels.

A within-group analysis to compare V2 and V3 values (e.g., fecal microbiota and salivary SIgA) will be conducted in each product group using a paired Student's *t*-test or a Wilcoxon signed-rank test, depending on the condition of application of the statistical test.

The outcomes related to GI symptoms and well-being (weekly average number of stools, stool form, amount of stools, color of stools, number of crying incidents, and duration of crying) will be analyzed using the following ANOVA model for repeated measurements (subject will be considered as a random effect, using SAS PROC MIXED):

Y = Product + Week + (Product × Week)

where Y = study endpoint at Wr (D8 to D15), W1 (D16 to D22), W2 (D23 to D29), W3 (D30 to D36), or W4 (D37 to D43)

Product = study products

Week = W1 to W4 weeks (between V2 and V3 visits)

Product × Week = interaction between the product and the week

### 2.4 Compliance analysis

Descriptive statistics on compliance will be performed. Compliance will be evaluated by the average of the daily compliance during the 4 weeks of the study. As the product will be consumed in the first feeding bottle of the day, parents will have to report if the bottle feeding is totally consumed and/or if there is any regurgitation.

Daily compliance will be calculated as follows:

((Volume of milk with study product drunk − Volume of milk regurgitated)/Volume of milk to be drunk) × 100

### 2.5 Safety analysis

All AEs will be listed, and descriptive statistics (imputability and intensity) of all the registered AEs will be provided by product. Infant growth endpoints (e.g., weight, height, and head circumference) will be analyzed using the same ANCOVA model used to analyze the primary endpoint. The proportion of subjects showing at least 1 AE will be compared between groups using a chi-Square or Fisher's exact test. The number of events will be compared between groups using a Poisson regression.

## 3 Results

### 3.1 Design and setting

This is a randomized, double-blind, placebo-controlled, 2-parallel-group trial, which will be conducted in the Shanghai First Maternity and Infant Hospital, Shanghai, China. Participating infants will undergo a 2-week run-in period and then be randomly allocated (at a 1:1 ratio) to receive either an oral probiotic product or an identical placebo once daily for 4 weeks, with a follow-up period of 8 weeks. Therefore, the duration of the subjects’ participation will be 14 weeks, with a total of 5 visits. Each parent who agrees to have his or her infant enrolled in the trial will sign an informed consent form; the study protocol has been approved by the Medical Ethics Committee of Shanghai First Maternity and Infant Hospital. The current study will be performed in accordance with the standards of the International Committee on Harmonization of Good Clinical Practice and the revised version of the Declaration of Helsinki.

### 3.2 Participant and eligibility

Two hundred infants, aged 4 to 6 months, will be included in this study. As no detailed data were available in the literature about the primary endpoint of the present study in the target population of interest, no formal sample size calculation has been performed. To meet the recommendations of the Chinese Center for Disease Control and Prevention (CCDC) (≥ 50 subjects per group) and anticipating a high drop-out rate, it has been decided to include 100 infants in each group of the study. The Committee on Nutrition of the European Society for Paediatric Gastroenterology, Hepatology, and Nutrition (ESPGHAN) has concluded that the administration of probiotic-supplemented infant formula during early life (≤ 4 months of age) does not result in any consistent clinical effects.^[20]^ The World Health Organization (WHO) recommends that infants start receiving complementary foods at the age of 6 months. Thus, the age of 4 to 6 months will be applied for the inclusion of subjects.

### 3.3 Inclusion criteria

Each infant will have to meet all of the following criteria to be enrolled in this study:

♦ Good health
♦ Single birth
♦ Gestational age ≥ 37 weeks (WHO)
♦ Birth weight ≥ 2500 g
♦ Appropriate weight at inclusion visit between P20 and P80 in accordance with the weight percentile charts of Chinese children aged 0 to 18 years^[21]^
♦ Aged > 4 months (120 days) and < 6 months (180 days)
♦ Being exclusively formula-fed (no breast milk meal) at the inclusion visit
♦ No gastrointestinal diseases within 1 month (CCDC)
♦ No use of antibiotics within 1 month (CCDC)
♦ Parents’ agreement to use one of the recommended infant formulas [no probiotics; if with prebiotics, galacto-oligosaccharides (GOS) < 2 g/100 g and no fructo-oligosaccharides (FOS) inside]
♦ Consent form signed by at least one of the parents or a legal guardian
♦ Parents able to understand the protocol requirements and to fill in the infant's diaries

### 3.4 Exclusion criteria

Infants meeting any of the following criteria will be excluded from the study:

♦ Congenital illness or malformation.
♦ Critical prenatal and/or postnatal disease.
♦ Mothers with metabolic and/or chronic disease.
♦ An allergic constitution and sensitive to probiotics (CCDC).
♦ Serious diseases, such as those of the cardiovascular, cerebrovascular, endocrine, liver, kidney, and hematopoietic systems, or mental diseases (CCDC).
♦ Other drugs during the administration of the study products, thus making it impossible to judge their efficacy and/or influencing the judgment of results (CCDC).
♦ Current or previous illnesses or conditions or interventions that could interfere with the study (affecting tolerance and/or growth), such as gastrointestinal malformations, chronic diarrhea, malabsorptive syndrome, malnutrition, congenital immunodeficiency, or major surgery, as per the investigator's clinical judgment.
♦ Oral antibiotic treatment at V1 visit and within 4 weeks before V1.
♦ Any medication or nutritional supplements (such as probiotics and prebiotics, except for infant formula) in the 4 weeks preceding the study start.
♦ Having ever consumed the test product.
♦ Medical conditions for which a special diet other than standard (nonhydrolyzed) cow's milk-based infant formula is required (such as cow's milk allergy, soy protein allergy, fish protein allergy, egg protein allergy, lactose intolerance, and galactosemia).
♦ Current participation or having participated in another clinical trial during the previous 4 weeks.
♦ Infants’ legal representatives without the psychological or linguistic capability to sign the informed consent form.
♦ Reasons to presume that the parents are unable to meet the study plan requirements (e.g., cannot contact the study representatives in the case of emergency, or have a drug addiction).

### 3.5 Randomization and blinding

The product allocation list will be generated using a dynamic randomization algorithm designed to minimize an imbalance between the 2 groups, taking stratification factors into account (method of delivery [vaginal route or cesarean delivery] and history of breastfeeding [breastfeeding present at the exit of maternity or not]). This allocation will be managed through the randomization management interface called the Interactive Web Response System (IWRS). All throughout the study, neither the investigator nor the subject will have access to the nature of the product they are testing (double-blind). The product labeling will not show any difference between active and placebo products. The randomization list (allocation of active and placebo batches) will be established by an independent person neither participating in the clinical phase nor processing the study data.

### 3.6 Study product and intervention

The study products, including active (probiotics) sachets and placebo sachets, are provided by Lallemand S.A.S., Blagnac, France. Each active (probiotics) sachet is 1.5 g, containing 1.425 × 10^8^ colony-forming units (CFU) of *Bifidobacterium infantis* R0033, 1.425 × 10^8^ CFU of *Bifidobacterium bifidum* R0071, and 9.6 × 10^9^ CFU of *Lactobacillus helveticus* R0052. Under refrigeration, the probiotics sachet is stable for 6 months at 2 to 4°C. The placebo sachet is made with 3% magnesium stearate and 97% potato starch, identically packaged and stored, and has the same appearance, color, and taste as the probiotics sachet. One sachet per day will be dissolved in the first feeding bottle of the day and then will be administered orally to participants by their parents or guardians for 4 weeks. If the participant vomits after taking the drug, no additional dose will be given. The volume of milk of the first feeding bottle actually drunk will be recorded in the case report forms (CRFs). The study products will be directly provided to parents according to the randomization list at the second visit (V2) in sufficient quantity for 28 days, as 28 sachets.

### 3.7 Study procedure and clinic schedule

**Figure 1:**
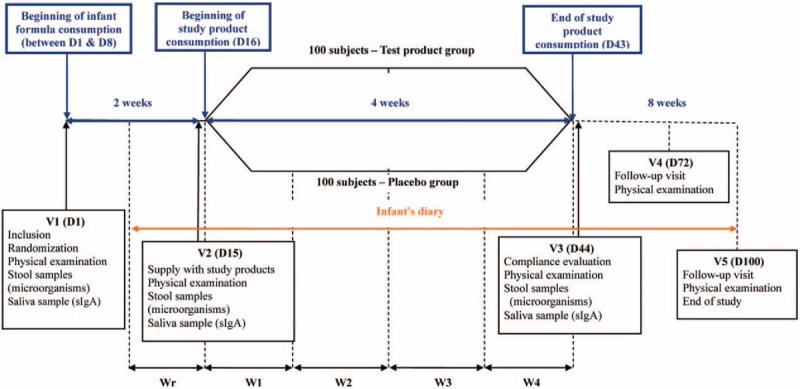
Flowchart of the trial.

**Figure 2:**
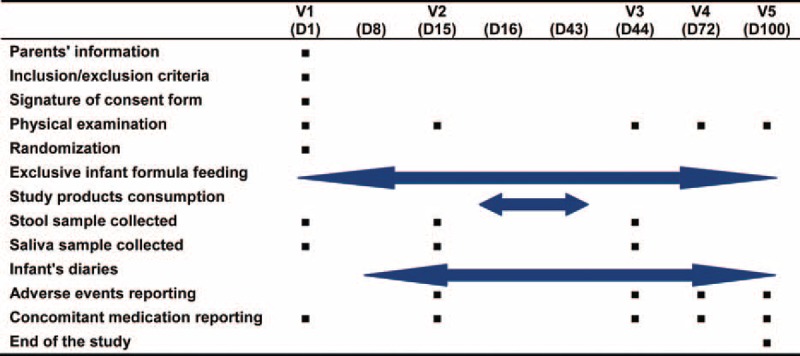
Clinic schedule of the trial.

Participants will be required to pursue a 2-week run-in, 4-week intervention, and 8-week follow-up period. The duration of subjects’ participation will be 14 weeks, with total 5 visits: inclusion (Visit 1, Day 1), start of intervention (V2, D15), end of intervention (V3, D44), and follow-up (V4 and V5, D72 and D100) (Figs. 1 and 2).

During the first visit (V1), a face-to-face interview will be conducted with the participant's parents. An investigator (pediatrician) will explain the study procedures in detail, particularly the purpose of the study, the procedures to be undergone, and the potential risks and benefits of participation. The inclusion and exclusion criteria will be reviewed to determine if the participant qualifies for the study. If the parents agree to have their infant enrolled in the trial, one of them will sign an informed consent. At the same time, the investigator will collect relevant information on demography, medical history, and concomitant medications.

During this first visit, parents can choose any brand of milk powder in the list of allowed infant formulas (without probiotics; if with prebiotics, GOS < 2 g/100 g and no FOS inside) presented by the investigators. The investigators will recommend the Biostime Infant Formula (Biostime Inc., Guangzhou, China). If parents are willing to choose the Biostime Infant Formula from the list, they will be provided a free and sufficient supply of the Biostime Infant Formula. The infant's feeding will have to be completely switched to the new infant formula within 1 week (D1-D8).

A saliva sample will be obtained at the site by the investigators at V1 (D1), V2 (D15), and V3 (D44). Parents will be instructed to collect a stool sample of their infant at home at V1 (before starting the new infant formula), V2 (within 24 h before the visit time), and V3.

The infant's diaries will be provided to parents at each visit, and they will be instructed to fill in these diaries daily from D8 to D99 in order to record GI symptoms (Bekkali scale),^[22]^ crying episodes (number and duration), number of stools, compliance (volume of milk of the first feeding bottle actually drunk, planned to be drunk, and regurgitated), and potential concomitant medications. Moreover, a physical examination will be conducted at each visit, and potential adverse events (AEs) will be monitored throughout the trial by the investigators.

### 3.8 Stool collection and bacteriological examination

Fresh stool samples will be collected in plastic bags, maintained anaerobically, and then sent to the lab for analysis. Stool samples (0.5 g) will be then mixed in 5 mL of sterile Ringer's solution containing 5% cysteine, allowed to vortex for 5 to 10 minutes, and shifted the supernatant for geometric dilution in a reagent bottle in sterile conditions.

Various media will be used for the selective isolation of different microorganisms: freshly prepared Man, Ragosa, and Sharpe (MRS) agar to which Mupirocin lithium salt is added, for *Bifidobacteria*; LBS agar supplemented with 1 mL/1000 mL of Tween 80 and 1.3 mL/1000 mL of glacial acetic acid, for *Lactobacilli*; TSC agar to which 0.8 mL/100 mL of 5% D-cycloserine is added, for *Clostridium perfringens*; modified GAM agar, to which 80 μg/mL of vancomycin, 1 μg/mL of kanamycin, 0.01 mL/100 mL of 1% vitamin K1, 0.25 g/100 mL of hemin, and 7 mL/100 mL of blood are added, for *Bacteroides*; bile esculin azide (BEA) agar, for *Enterococci*; and violet red bile dextrose (VRBD) agar, for *Enterobacteria*.

The seeded, streaked culture plates will be placed in anaerobic containers to maintain anaerobic conditions and then will be incubated at 37°C. Each anaerobic container contains a resazurin oxygen reduction indicator to ensure *anaerobicity* during incubation. The MRS, LBS, and modified GAM plates will be incubated anaerobically for 48 hours; the BEA, TSC, and VRBD agar plates will be incubated anaerobically for 24 hours. Colony counts will be done manually and expressed as CFU/g; numbers over 300 will be designated as too numerous to count (TNTC).

After incubation, colonies with representative morphology and color typical of specific species will be randomly selected and identified with the microorganism MASS system for confirmation.

### 3.9 Saliva collection and SIgA measurement

Infants will be allowed to chew or suck on the swab for 30 to 60 seconds, followed by mopping up any pooled saliva left in the mouth or on the face. The samples are then recovered immediately by compression in a syringe, and the procedure is repeated to collect more saliva if the initial amount is too small.

Saliva samples will be centrifuged at 13 000 × g for 5 minutes at room temperature, and then the supernatant is stored at −20°C. For the SIgA assay, the SIgA concentration is assessed by means of particle-enhanced immunonephelometry. Briefly, the saliva samples are processed at a 1:5 dilution (as recommended), and evaluation of SIgA is performed automatically in mg/L. Reference curves are generated by multi-point calibration. Serial dilutions of N IgA Standard (human) are automatically prepared by the instrument using N Diluent.

### 3.10 Safety assessment

An adverse event (AE) has been defined as any unfavorable or unintended clinical or other occurrence during the study period that may or may not be causally related to the intervention. All AEs considered not, unlikely, possibly, probably, or definitely related to the test product will be recorded in the CRFs.

A serious AE is defined as event that is fatal, life-threatening, disabling, or incapacitating or results in hospitalization, a prolonged hospital stay, or malformation. All will be also recorded in the CRFs, whether they are related to the test product or not. Probiotics are generally considered safe and well tolerated, and any serious AEs that might be possibly, probably, or definitely related to the test product will be regarded as unexpected. All serious AEs will be reported as soon as possible to the national Food and Drug Administration (FDA) and local FDA, the sponsor, and Infant Hospital. Any serious AEs that might be related to the test product will immediately lead to the discontinuation of the test product, and the study subject will be followed until the conclusion of the event.

### 3.11 Quality control

To maintain the accuracy and quality of the clinical trial, audits and monitoring will be implemented by the sponsor. Clinical research associates will regularly monitor whether the clinical trial is proceeding based on the protocol by checking trial master files, informed consent forms, CRFs, adverse events, and compliance with study productions.

### 3.12 Study endpoint

The primary endpoints are the V2-V3 changes in composition of the fecal microbiota, including *Bifidobacterium bifidum*, *Bifidobacterium longum ssp. infantis*, *Lactobacillus helveticus*, *Clostridium perfringens*, *Enterococcus*, *Enterobacteria*, and *Bacteroides* (defined as V3 [D44]-V2 [D15]). The secondary endpoints are:

♦ the V2-V3 change in SIgA antibody levels in saliva samples
♦ GI symptoms and well-being, as recorded daily by parents in the infant diaries; the following outcomes will be calculated on a weekly basis:
  1. average daily number of stools; for each subject, the total number of stools per day will be recorded in a 7-day diary, and then the average daily number of stools will be calculated. (The same procedure will be applied for the following endpoints.)
  2. average daily Infant Stool Form scores consistency (4-point scale), average weekly amount (4-point scale, letters will be replaced by figures, i.e., “A” to “1,” “B” to “2,” etc.) and color (6 categories), using the validated scale for preterm and term infants proposed by Bekkali et al.^[22]^
  3. average daily number of crying episodes
  4. average daily duration of crying episodes.
♦ the changes in growth parameters such as weight, length, and head circumference
♦ the numbers of AEs and serious AEs

## 4 Discussion

In the early stage of life, the composition of the intestinal microbiota undergoes major modifications, mostly influenced by feeding patterns.^[23]^ Intestinal microflora composition differs substantially in breast-fed infants and formula-fed infants because of the differences in composition between human milk and standard infant formula. The breast-fed infant's microbiota is composed of an increased number of *Bifidobacteria* and *Lactobacilli*, whereas the formula-fed infant's microbiota has more *Enterococci* and *Enterobacteria.*^[24,25]^ It is widely accepted that breast-fed infants are often healthier than formula-fed infants and can fight infections better. This difference is considered to be due to breast milk's composition with molecules with antimicrobial activity and prebiotic oligosaccharides with various health benefits.^[26,27]^ In fact, healthy intestinal microflora are essential to infants’ health, and the composition of intestinal microflora could be adapted by consuming probiotics in infancy.^[28]^ Therefore, the first 2 to 3 years of life are the most critical period in which one can intervene to shape the microbiota as best as possible, and so optimize child growth and development.

Numerous randomized controlled trials have shown that the use of probiotic products, including those that are added to commercially available infant formula and other food products for children, is effective in preventing and treating common diseases such as diarrhea and allergy.^[29,30]^ However, little is known about whether probiotics could offer benefits to healthy infants. With the boom of probiotics appearing on the market, the use of probiotic products in the community is becoming more widespread.^[31]^ It is therefore important to provide sufficient scientific evidence for its effectiveness in the digestibility and immunity of exclusively formula-fed healthy infants. An effective, practical, and acceptable intervention for these infants would represent a major clinical and public health advance. If cost effective, the simplicity of the intervention is such that the administration of probiotic supplementation or follow-on formula to infants, given beyond early infancy, may be associated with some clinic benefits. However, it is worth noting that despite there currently being no safety concerns regarding probiotics, more research is required regarding the long-term and routine use of probiotic-supplemented formula to healthy infants.
